# Supplementary material for: Assortative mating frames establishment in a young island bird population
Source: R Soc Open Sci. 2019 Aug 14;6(8):190050. doi: 10.1098/rsos.190050 (PMC6731715; doi:10.1098/rsos.190050)
Supplement: Supplement S1: DNA Extraction and Genotyping and Table S1 [file rsos190050supp1.docx]

# Assortative mating frames establishment in a young island bird population

**Jan O. Engler^1*^, Thomas Sacher^2,3^ , Timothy Coppack^2,4^, Franz Bairlein^2^**^1^ Department of Biology, Terrestrial Ecology Unit, Ghent University, 9000 Ghent, Belgium

^2^ Institute of Avian Research, Vogelwarte Helgoland, D-26386 Wilhelmshaven, Germany

^3^  *Current address:* Im Mühltal 33, 61203 Reichelsheim, Germany

^4^  *Current address:* Mägdebrink 5, 37574 Einbeck, Germany

*corresponding author, email: JanOliver.Engler@ugent.be, twitter: @engler_j

## Supplement S1: DNA Extraction and Genotyping

We genotyped the entire set of 822 individuals taken from blood or feather samples using seven polymorph microsatellites (Table S1) and preserved samples in 96% ethanol before extraction. We used the Qiagen DNeasy Blood and Tissue kit (Quiagen, Hilden, Germany) for DNA extraction by following the manufacturers protocol. Next to this we estimated DNA concentrations in ten samples using a Versaflour Flurometer System (Bio-Rad Laboratories) using the Fluorescent DNA quantification kit by Hoechst. The average DNA concentration was 40 ng/µl (range 1 - 70 ng/µl). PCR amplifications were conducted using 10 µl reactions (see [1] for details) in two multiplex runs at a constant annealing temperature of 56°C (Table 1). Conditions for PCR reactions were setted by an 2 min denaturation step (94°C) followed by 35 cycles of 30 s denaturation (94°C), 30 s annealing at 56°C and 30 s extension (68°C) and finalized by 5 min at 68°C. PCR electrophoresis was performed on an ABI 377 sequencer using a Genescan-500 (LIZ) size standard (Applied Biosystems). Subsequent genotyping was done with Genescan Analysis v 3.7. and Genotyper v. 3.7. (Applied Biosystems).
We used PopGenReport in R 3.3.1 [2] to estimate locus-specific null allele frequencies, and FSTAT 2.9.3.2. [3] to test for linkage disequilibrium. No linkage disequilibria among loci could be found and null allele probabilities were generally low (i.e. < 0.05, Table S1) so that we kept all loci for the subsequent analyses. 

1. Segelbacher G, Sacher T, Schwarzenberger A, Woitsch S, Bairlein F, Coppack T. 2007 Eight microsatellite loci characterised in the European blackbird *Turdus merula*. J Ornithol 149**:** 131 – 133. (doi:10.1007/s10336-007-0227-0)
2. Adamack AT, Gruber B 2014: PopGenReport: simplifying basic population genetic analyses in R. Methods Ecol Evol 5: 384-387.
3. J Goudet. FSTAT, a program to estimate and test gene diversities and fixation indices (version 2.9. 3). (2001).
4. Kudernatsch D, Weis-Dootz J, Segelbacher G. 2009 Isolation of ten tetranucleotide microsatellite loci in the Northern Wheatear (*Oenanthe oenanthe*). Mol Ecol Res 9: 542-543. (doi:10.1111/j-1755-0998.2008.02341.x)
5. Gibbs HL, Tabak LM, Hobson K. 1999 Characterization of microsatellite DNA loci for a neotropical migrant songbird, the Swainson’s thrush (*Catharus ustulatus*). Mol Ecol 8: 1551 – 1552.
6. Simeoni M, Dawson DA, Gentle L, Coiffait L, Wolff K, Evans KL, Gaston K, Hatchwell BJ. 2009 Characterization of 38 microsatellite loci in the blackbirds *Turdus merula* (Turdidae, AVES). Mol Ecol Res 9: 1520 – 1526.

**Table S1: Summary of the used microsatellite loci for genotyping European blackbirds.** *n* refers to the number of genotyped individuals, *N_a_* to the number of alleles, *H_o_* and *H_e_* to observed and expected heterozygosities, *F_is_* to the inbreeding coefficient, *F_st_* to the fixation index, *D* to linkage disequilibrium probability, and *0a* to null allele frequency.

| **Locus** | **EMBL accession number** | **Reference** | **Multiplex set** | **Dye label** | **n** | **Allele size (bp)** | **N**_a_ | **H**_o_ | **H**_e_ | **F**_is_ | **F**_st_ | **D** | **0a** |
| --- | --- | --- | --- | --- | --- | --- | --- | --- | --- | --- | --- | --- | --- |
| Oe1 | EU573949 | [1] | 1 | 6-FAM | 806 | 142-199 | 20 | 0.758 | 0.738 | -0.009 | 0.028 | 0.064 | 0.011 |
| Oe7 | EU573954 | [4] | 1 | NED | 822 | 170-197 | 9 | 0.691 | 0.64 | 0.013 | 0.053 | 0.09 | 0.031 |
| tur03 | EU573963 | [1] | 1 | VIC | 822 | 163-205 | 13 | 0.78 | 0.726 | 0.016 | 0.063 | 0.179 | 0.03 |
| Cu32 | AF122895 | [5] | 2 | VIC | 819 | 153-188 | 19 | 0.839 | 0.772 | -0.005 | 0.039 | 0.16 | 0.038 |
| LTMR6 | FM201465 | [6] | 2 | 6-FAM | 821 | 212-242 | 21 | 0.83 | 0.781 | 0.011 | 0.027 | 0.087 | 0.027 |
| tur02 | EU573962 | [1] | 2 | NED | 822 | 178-212 | 14 | 0.785 | 0.781 | -0.068 | 0.045 | 0.131 | 0.002 |
| tur01 | EU573961 | [1] | 2 | 6-FAM | 821 | 163-171 | 3 | 0.474 | 0.417 | 0.014 | 0.083 | 0.077 | 0.04 |
