# Supplementary material for: Assortative mating frames establishment in a young island bird population
Source: R Soc Open Sci. 2019 Aug 14;6(8):190050. doi: 10.1098/rsos.190050 (PMC6731715; doi:10.1098/rsos.190050)
Supplement: Supplement S2: in silico admixture of Northern and Central European genotypes [file rsos190050supp2.docx]

# Assortative mating frames establishment in a young island bird population

**Jan O. Engler^1*^, Thomas Sacher^2,3^ , Timothy Coppack^2,4^, Franz Bairlein^2^**^1^ Department of Biology, Terrestrial Ecology Unit, Ghent University, 9000 Ghent, Belgium

^2^ Institute of Avian Research, Vogelwarte Helgoland, D-26386 Wilhelmshaven, Germany

^3^  *Current address:* Im Mühltal 33, 61203 Reichelsheim, Germany

^4^  *Current address:* Mägdebrink 5, 37574 Einbeck, Germany

*corresponding author, email: JanOliver.Engler@ugent.be, twitter: @engler_j

## Supplement S2: *in silico* admixture of Northern and Central European genotypes

To better understand observed genetic patterns of the island population and linking them to colonization and local recruitment, we composed a set of simulations based on the available genotypic information from non-island birds. In a first scenario, we assumed that colonization was entirely driven by migrating individuals which stayed on the island. In a second scenario, we assumed colonizing individuals arrived exclusively from nearby mainland populations. In a third scenario, we assumed that both migrants and mainland genotypes contribute equally to the colonization. For the ease of illustration, we generated 100 *in silico* recruits out of the allele frequencies available under each scenario assuming random mating (i.e. from migrants in Scenario 1, from mainland locations in Scenario 2, and from an equal contribution of both in Scenario 3). We then used Structure to assess population structures of the *in silico* genotypes from each simulation run together with genotypes of nestlings from the island. Structure settings were the same as those used for the empirical data except that we used a fixed *K* at the same value (i.e. K = 2) as has been identified using the observed data. We then compared individual as well as group-averaged assignments to *K* among the simulation runs and the observed values of the island nestlings using the *dabestr* package in R [1]. In particular, we calculated the unpaired mean difference between the empirical reference (i.e. the island nestlings) values of the assignment probabilities for the first cluster. The simulation runs showed diverging patterns of individual cluster assignment. Under Scenario 1 expecting island colonization by migrant individuals, we found a clear clustering of *in silico* recruits to the cluster corresponding to the genotyped migrant birds (unpaired mean difference: -0.869, 95%CI: -0.903 to -0.825). This coincides with the generally high frequency of genotypes belonging to this cluster in migrants. In contrast, Scenario 2 that expected a mainland origin showed a diverging picture with a high number of simulated individuals showing a high amount of admixture thus failing to clearly assign to one of the two clusters. Yet the mean difference came closest to the observed situation (unpaired mean difference: -0.22, 95%CI: -0.261 to -0.177). This pattern of high admixture became even stronger for Scenario 3 that assumed a mixed origin of island birds (unpaired mean difference: -0.523, 95%CI: -0.556 to -0.486). In sum, none of the three scenarios conducted resulted in a pattern that is comparable to the clearly distinct distribution of genotyped island nestlings with generally low admixture.  

1. Ho J, Tumkaya T, Aryal S, Choi H, Claridge-Chang A. 2018 Moving beyond p values: Everyday data analysis with estimation plots. *bioRxiv* (doi:10.1101/377978)

*
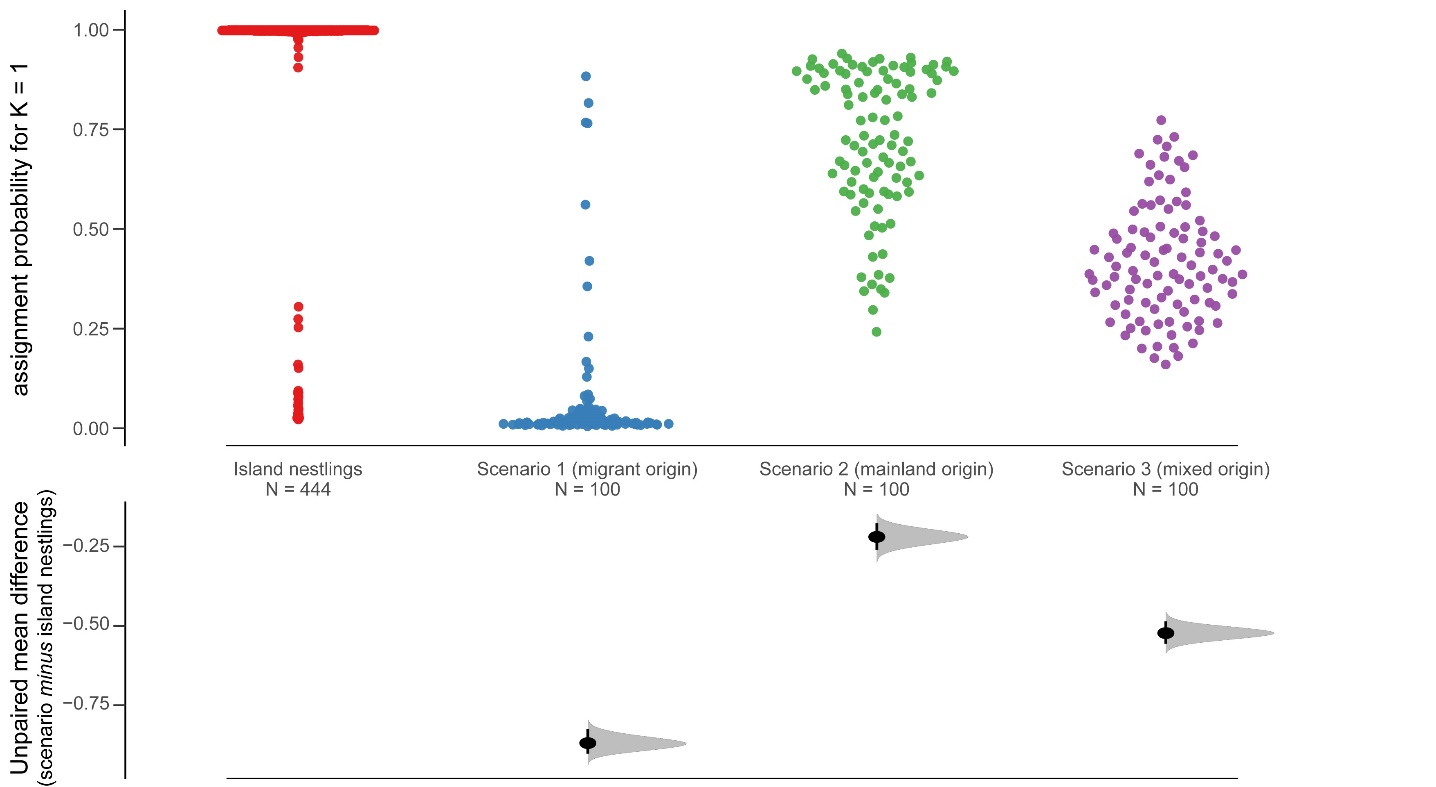
*

**Fig. S1: Distribution of assignment values for genotyped island nestlings (red) and simulated recruits under three different scenarios (blue, green, purple).** The lower panel shows the mean and 95% CI unpaired mean difference with its underlying distribution that is based on 5000 bootstraps. In observed island nestlings, assignments predominantly cluster to one group and to a lesser extent to the second group with just a few intermixed (i.e. hybrid) individuals. In contrast, simulations based on available allele frequencies from different source scenarios lead to a much higher amount of admixture.
